# Determination of the Best Controlled-Release Potassium Chloride and Fulvic Acid Rates for an Optimum Cotton Yield and Soil Available Potassium

**DOI:** 10.3389/fpls.2020.562335

**Published:** 2020-11-17

**Authors:** Jibiao Geng, Xiuyi Yang, Xianqi Huo, Jianqiu Chen, Shutong Lei, Hui Li, Ying Lang, Qianjin Liu

**Affiliations:** ^1^ Shandong Provincial Key Laboratory of Water and Soil Conservation and Environmental Protection, State Key Laboratory of Nutrition Resources Integrated Utilization, College of Agriculture and Forestry Science/Resources and Environment, Linyi University, Linyi, China; ^2^ Kingenta Ecological Engineering Group Co., Ltd., Linshu, China

**Keywords:** fulvic acid, controlled-release potassium chloride, cotton yield, leaf photosynthesis, soil available potassium, net profit

## Abstract

Potassium and fulvic acid (FA) fertilizer applications are two important measures for improving cotton growth. However, there are few studies on the application interactive effects of controlled-release potassium chloride (CRK) in combination with FA on cotton production. To explore the effects of CRK combined with FA on cotton, field experiments were conducted in 2018 and 2019 using a split-plot design. The main plots were assigned to two types of potassium fertilizer – controlled-release potassium chloride (CRK) and potassium sulfate (KS) – while low, moderate, and high FA application rates (90, 180, and 270 kg ha^−1^) were assigned to the subplots. The cotton yield, fiber quality, net profit, soil available potassium concentration, potassium use efficiency, and leaf photosynthesis were markedly affected by potassium fertilizer and FA. The cotton boll number and boll weight in the 2 years and the yield in 2019 were all affected by the interaction between potassium fertilizer and FA. Compared to the other potassium treatments, the CRK × FA180 treatment increased the seed yield and net profit by 4.29–14.92% and 13.72–62.30%, respectively, over the 2 years. The potassium agronomy efficiency and potassium recovery efficiency (KRE) of the CRK × FA180 treatment were also improved by 6.25–30.77% and 3.82–12.78% compared to those of the other potassium treatments. Overall, the FA180 treatment resulted in better cotton growth than that in the FA90 and FA270 treatments. The release period of CRK in the field during the growth period of cotton was longer than that detected by 25°C static water extraction, which increased the soil available potassium content and met the potassium demands over the whole cotton growth period. Therefore, the application of CRK in combination with 180 kg ha^−1^ FA is the best choice for cotton fertilization.

## Introduction

At present, China is the largest consumer and producer of cotton in the world, and the demand for cotton production and quality is constantly increasing (Feng et al., 2017). Cotton exhibits a high potassium demand, and potassium nutrition plays a key role in the determination of the cotton yield and fiber quality (Hatam et al., 2020). Potassium is an important element for crop growth and development that plays a vital role in maintaining the cell osmotic pressure balance (Zahoor et al., 2017), improving the stomatal movement, ensuring enzyme activity, optimizing the photosynthetic performance, promoting the transport of assimilates (Hafeez et al., 2018), and improving plant resistance to biotic and abiotic stresses (Shahzad et al., 2019). In addition, potassium can improve the utilization rate of nitrogen fertilizer, promote the growth of roots, stems, leaves, and reproductive organs of cotton plants, prolong the functional period of leaves, and prevent premature aging (Hu et al., 2017).

There is a general lack of potassium in China’s cultivated land, and the total area of seriously deficient (available potassium < 50 mg kg^−1^) and moderately deficient (available potassium of 50–70 mg kg^−1^) land is more than 22.67 million ha, accounting for 22.6% of the total cultivated land area (Chen et al., 2018). In recent years, with the increase in cotton yield, the application of high-yield varieties, and the increase in nitrogen and phosphorus fertilizer, the removal of soil potassium by cotton has increased year by year, and the loss of soil potassium from cotton fields cannot be effectively supplemented (Yin et al., 2018). In some areas, the content of soil available potassium has decreased, and potassium deficiency has been observed.

Potassium fertilizer application is the main measure by which farmers supplement soil potassium. However, the potash fertilizer resources in China are very low. The domestic production of potash fertilizer has been reported to be 3.774 million tons, and the import dependence of potash fertilizer as high as 50% (Zheng et al., 2016a). Furthermore, traditionally available potassium fertilizers, such as potassium chloride and potassium sulfate, are easily fixed or transformed into non-exchangeable and fixed potassium with a low effectiveness in the soil or are leached by rainwater or lost through surface runoff, resulting in the early decline of cotton due to potassium deficiency at later stages (Jia et al., 2016; Tian et al., 2017; Chen et al., 2020). The large growth habit of cotton plants and their many fruit branches lead to high labor costs and low incomes from cotton planting with potassium fertilizer topdressing. Therefore, it is of great significance to study reasonable fertilization measures to improve the yield of cotton and realize efficient and simplified cotton production.

With the continuous development and improvement of controlled-release fertilizers, research on controlled-release potassium fertilizer for crops is increasing (Yang et al., 2016, 2017; Li et al., 2020). Controlled-release potassium fertilizer, especially controlled-release potassium chloride (CRK), has become a research hotspot. CRK can release nutrients slowly through a polymer coating according to the characteristics of the crop fertilizer demand and can therefore meet the demand for potassium during the growth and development of crops (Chen et al., 2020).

Humic acid (HA) has become a popular new fertilizer in recent years. It is a kind of natural organic polymer mixture formed by the decomposition and transformation of animal and plant debris with the participation of microorganisms and a series of geochemical processes (Sehaqui et al., 2017). In areas, where HA fertilizer is applied, the advantages of HA have been fully proven. First, HA loosens the soil and improves the soil fertility. Second, it improves the utilization rate of fertilizer and reduces the loss of nutrients. Third, it strengthens the activities of various enzymes in the plant to stimulate the growth of crops. Fourth, it promotes the propagation and activity of microorganisms such as fungi and bacteria and increases the activity of nitrogen-fixing bacteria, which accelerates the decomposition of organic matter, accelerates the maturity of agricultural fertilizer, and promotes the release of available nutrients (Selladurai and Purakayastha, 2016; Li et al., 2019; Shahbazi et al., 2019).

Fulvic acid (FA) is an organic aromatic substance with a small fraction size and high activity, and it is one of the effective components of HA (Mahoney et al., 2016). FA is a component of HA, so it has the general characteristics of HA; however, because it has other characteristics not possessed by HA, it has attracted the attention of international soil scientists, chemists, coal chemists, and plant physiologists (Moradi et al., 2017). There are some differences between FA and HA. FA has lower molecular weight and is easier to be absorbed than HA. Its functional group content makes it have higher physiological activity than HA, and has strong complexation ability with metal ions. HA is not directly soluble in water, so it needs to be converted into monovalent metal salts such as potassium, sodium, or salt, whose aqueous solution is alkaline, but FA can be directly soluble in water and its aqueous solution is acidic (Ahmad et al., 2018; Khan et al., 2019).

Many studies have been carried out on the effects of CRK or FA fertilizers on cotton growth, yield, and nutrient absorption, but most have focused on a narrative discussion or a single verification of the effects of the fertilizer on cotton (Tian et al., 2017; Yang et al., 2017). There are few studies on the interactive application effects of CRK in combination with FA on cotton production. It was hypothesized that the interaction in the application of CRK and FA would enhance the cotton yield, soil available potassium, and potassium use efficiency. Hence, the objective of this study was to investigate the effects of CRK combined with FA on the (i) cotton leaf senescence, (ii) potassium use efficiency, (iii) soil available potassium, and (iv) cotton yield and economic benefit.

## Materials and Methods

### Experimental Materials

**Figure 1:**
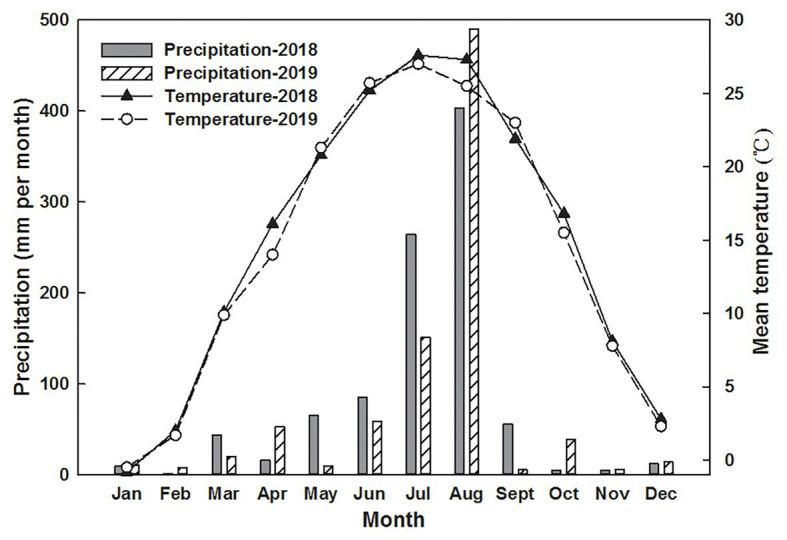
Air temperature and precipitation in 2018 and 2019.

The experimental site was in Niujiaxiaohe village, Linyi city, Shandong Province, China (N 35°48'33''; E 118°26'45''), in 2018 and 2019. This area has a temperate monsoon climate, with rainfall concentrated from July to September. The mean temperatures during the growth season (May to October) were 23.26 and 23°C, and the total precipitation was 878.5 and 754 mm in 2018 and 2019, respectively (Figure 1). The tested arable soil contained 18.5% clay, 16.8% sand, and 64.7% silt, which constitutes a silty clay loam. The soil type was classified as Typic Hapludalf according to the USDA classification method (Soil Survey Staff, 1999). The pH value was 6.71, and the organic matter, total N, NO_3_^−^-N, NH_4_^+^-N, available phosphorus, and available potassium contents were 6.8, 0.82, 56.33, 24.14, 36.22, and 130.07 mg kg^−1^, respectively. Cotton variety “Lumianyan 28” was used, and the planting density was 50,000 plants ha^−1^.

The tested fertilizers included CRK and other traditional fertilizers. The production process of CRK (containing K_2_O 55%) was as follows. First, potassium chloride powder was poured into a disk granulator for granulation, and 3–5 mm particles were screened out for drying. After the surface was uniform and smooth, it was coated with epoxy resin at a coating thickness of 5%. The other traditional fertilizers included FA (pure FA content 50%), urea (N 46%), potassium sulfate (KS; K_2_O 50%), and calcium superphosphate (P_2_O_5_ 14%).

### Experimental Design

The experiment used a split-plot design with triplicate-duplicate partition. The control was a no-potassium treatment. The potassium types (CRK, KS) defined the main plots, and the FA rates (90, 180, and 270 kg ha^−1^) defined the subplots. The main plot covered an area of 90 m^2^ (5 m wide and 18 m long), and the subplot covered an area of 30 m^2^ (5 m wide and 6 m long). The N, P_2_O_5_, and K_2_O application amounts were 180-90-180 kg ha^−1^. All fertilizers were applied once by hand before sowing. Other agronomic management measures were implemented according to local agronomic practices.

Before the experiment, nylon mesh bags with a width of 8 cm and a length of 10 cm were made. CRK particles (10 g) were weighed and placed into the mesh bags, and the bags were sealed. In the CRK treatment area, a ditch (8 cm deep and 12 cm wide) was dug 10 cm away from one side of the sowing row, and 30 mesh fertilizer bags were laid on the bottom of the ditch. The fertilizer particles in the mesh bags were evenly spread to cover the soil in the ditch to determine the release characteristics of CRK into the field soil (10–15 cm deep).

### Sampling and Measurement

According to previous experience, there is no significant difference in the values of some indicators in the first year as the advantages of controlled-release KCl fertilizers increase with time. Consequently, it is more meaningful to present data from the second year of the study (2019).

Cotton was sown on 26 April 2018 and 28 April 2019. Soil and plant samples were collected at the bud stage (52 days after sowing), the early flowering stage (76 days after sowing), the full boll-setting stage (93 days after sowing), the initial boll-opening stage (118 days after sowing), and the harvest stage (195 days after sowing) in 2019.

### Soil Sampling and Measurement

Soil samples at 0–20 cm were collected in a five-point sampling pattern with a soil drill (two sampling points in the fertilizer row, two sampling points in the cotton row, and one sampling point in the unplanted row). After mixing, the samples were taken back to the laboratory for air drying and passed through a 10-mesh sieve. The contents of NO_3_^−^-N and NH_4_^+^-N in some of the fresh soil (0.01 mol L^−1^ CaCl_2_ extraction) were determined immediately using an AA3 continuous flow analyzer (Bran-Luebbe, Norderstedt, Germany). The remaining soil was air-dried and ground through 2 and 0.25 mm sieves, and the organic matter (potassium dichromate external heating method), soil total N (semi-micro Kjeldahl method), available phosphorus (pH 8.5, 0.5 mol L^−1^ NaHCO_3_ extraction, molybdenum blue colorimetry) and available potassium content (1 mol L^−1^ NH_4_OAc extraction, flame photometer method) were determined (Zheng et al., 2016b).

### Photosynthetic Parameters of Cotton Leaves

At the full boll-setting stage under sunny and cloudless weather from 9:00 to 10:00 AM, the photosynthetic parameters of fully expanded leaves (fourth main-stem leaf from the apex) were determined using three randomly selected plants in the central two rows of each plot. SPAD-502 readings were used as a proxy of the chlorophyll content of leaves (SPAD-502, Minolta, Japan). A Li-6400 portable photosynthetic apparatus (LI-COR, Lincoln, NE, United States) was also used to measure the net photosynthetic rate (*P*_n_), stomatal conductance (*G*_s_), intercellular carbon dioxide concentration (*C*_i_), and transpiration rate (*T*_r_). The fluorescence parameters of chlorophyll, including the primary light energy conversion efficiency (*F*_v_/*F*_m_), non-photochemical quenching coefficient (*q*_N_), photochemical quenching coefficient (*q*_P_), and effective quantum yield of PSII photochemistry (*Φ*PSII), were measured by an FMS2 portable fluorescence system (Hansatech instruments, King’s Lynn, Norfolk, United Kingdom). The *F*_v_/*F*_m_ determination required leaves to be dark-adapted for half an hour (Song et al., 2019).

### Cotton Yield and Fiber Quality

At harvest, each plot and the number of bolls of 20 consecutive plants were recorded. One hundred of those bolls were chosen randomly, oven dried, and weighed to determine the mean boll weights. The cotton lint percentage was measured after ginning with a roller. The cotton yield was calculated according to the number of bolls, the single boll weight, and the lint content. The fiber quality was determined by the cotton quality supervision, inspection, and testing center of the Ministry of Agriculture (Anyang, Henan Province, China).

### Plant Sampling and Measurement

Five cotton plant samples were randomly collected during the harvest period. The collected plants were separated, mixed, dried, weighed, and crushed by organ (stem, leaf, fiber, seed, and boll shell). The mass of dry matter in each organ was obtained by killing at 105°C for 30 min and then drying at 80°C to a constant weight. The sum of the dry matter of each organ yielded the total mass of the dry matter. The potassium contents of the different organs of the plants were analyzed. The aboveground potassium uptake was calculated based on the potassium content and dry matter quality of each part. The potassium content of the plant was determined through H_2_SO_4_-H_2_O digestion and flame photometry. Finally, the potassium recovery efficiency (KRE) and potassium agronomic efficiency (KAE) were calculated (Chen et al., 2020).

### Determination of the CRK Release Rate

Controlled-release potassium chloride was released in still water at 25°C and determined according to the determination method of controlled-release fertilizer in the chemical industry standard of the People’s Republic of China “Hg/T 4215-2011” (Li et al., 2016). The release of CRK into the soil was determined by the bag burial-weighing method in 2019. The procedure involved weighing 10 g of the CRK particles into nylon mesh bags with a length of 10 cm and width of 8 cm and burying them in a cement tank with a depth of 15–20 cm during fertilization. The net bags were collected on the 10, 20, 30, 60, 90, 120, and 180th days after burying the bags. Three bags were collected each time. The soil on the surface of the fertilizer particles was washed and dried to a constant weight at 60°C, and the potassium release rate was calculated according to the quality of the remaining fertilizer particles. The release of CRK was considered complete when the potassium content in the remaining particles reached 80%.

### Statistical Analyses

The data were analyzed using SAS software (version 10, SAS Institute Cary, NC, United States), and figures were drawn in SigmaPlot software (version 12, MMIV, Systat Software Inc., San Jose, CA, United States). The data presented are the average values of three repetitions.

## Results

### Release Rate of CRK

**Figure 2:**
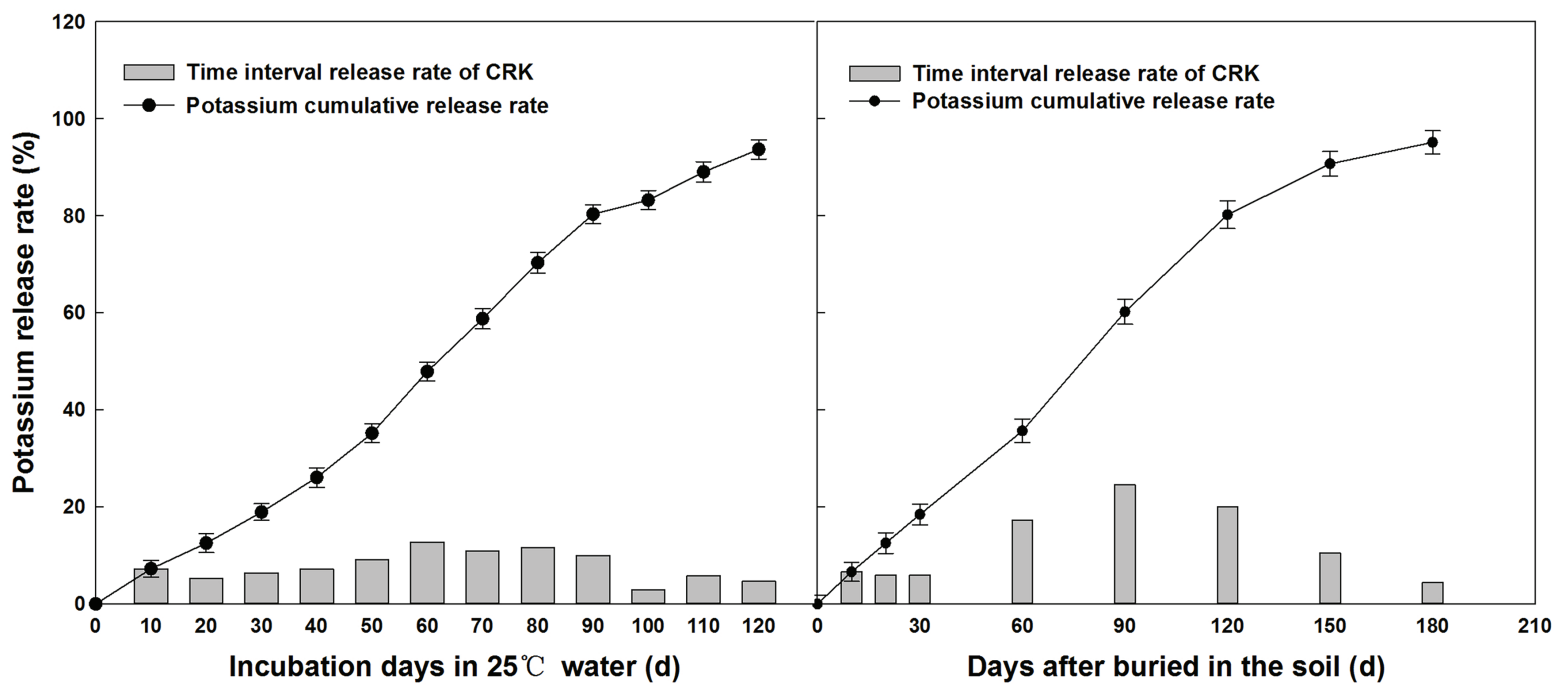
Release of potassium from controlled-release potassium chloride (CRK).

**Figure 3:**
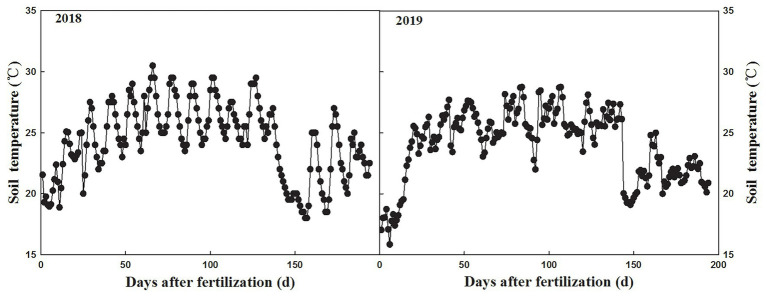
Soil temperature in 2018 and 2019.

The potassium nutrient release from the CRK in static water at 25°C was plotted as a straight line (Figure 2). The CRK release showed a trend of slow, fast, and then slow. More than 80% of potassium nutrients were released in approximately 90 days. The release characteristics of the CRK in field soil were similar to those in 25°C water, but the release time was longer, reaching 80% in 120 days. The average soil temperature was 24.36°C (Figure 3) after fertilization, which was lower than that in the laboratory (25°C water). The release period of CRK was almost 180 days. The release rate was slow within 10–60 days and increased within 60–120 days, and the nutrient decline period was 120–180 days. Therefore, CRK met the potassium demands of cotton through the whole growth period.

### Cotton Leaf Photosynthesis

**Figure 4:**
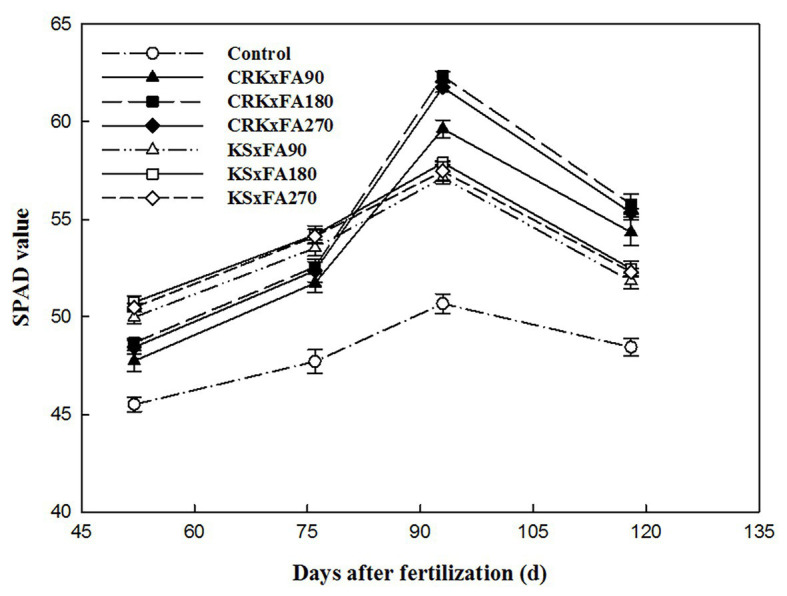
SPAD values of cotton leaves.

From the bud stage to the full boll-setting stage, the SPAD value of every treatment increased and then decreased gradually from the initial boll-opening stage, and the SPAD value of the control treatment was the lowest (Figure 4). For all potassium fertilizer types, the SPAD value increased first and then decreased with increasing FA rates, and the SPAD value of the medium FA rate treatment (FA180) was the highest. In addition, the SPAD values of the KS treatments were higher than those of the CRK treatments from the bud stage to the early flowering stage. However, the SPAD values of the CRK treatments were significantly higher than those of the KS treatments from the early flowering stage to the initial boll-opening stage. The SPAD value of CRK × FA180 was the highest among these treatments.

The potassium fertilizer type and the FA rate affected the photosynthesis and chlorophyll fluorescence indicators, but there was no significant difference between their interactive effects (Table 1). Specifically, the net photosynthetic rate (*P*_n_), stomatal conductance (*G*_s_), and transpiration rate (*T*_r_) were higher, but the intercellular carbon dioxide concentration (*C*_i_) of the FA180 treatments was lower than those of the FA90 and FA270 treatments. The FA180 treatments also improved the effective quantum yield of PSII photochemistry (*Φ*PSII), the light conversion efficiency of PSII in the dark (*F*_v_/*F*_m_), and the coefficient of photochemical quenching (*q*_P_) rates, but the coefficients of non-photochemical quenching (*q*_N_) were lower than those of the FA90 and FA270 treatments. Similarly, the photosynthetic and fluorescence indexes of the CRF treatments were significantly higher than those of the KS treatments. In conclusion, CRK × FA180 significantly improved photosynthesis in cotton leaves.

**Table 1:** Parameters of the photosynthetic chlorophyll and fluorescence of cotton leaves at the full boll-setting stage, 2019.

Treatment	*P*_n_	*G*_s_	*T*_r_	*C*_i_	*Φ*PSII	*F*_v_/*F*_m_	*q*_P_	*q*_N_
	(umol m^−2^ s^−1^)	(umol mol^−1^)	(umol m^−2^ s^−1^)	(umol m^−2^ s^−1^)				
Potassium fertilizer type								
CRK	22.21 a	0.66 a	11.63 a	267.02 b	0.65 a	0.86 a	0.95 a	1.01 b
KS	18.95 b	0.59 b	10.76 b	278.09 a	0.61 b	0.81 b	0.89 b	1.09 a
Fulvic acid (FA) rate (kg ha^−1^)								
90	20.19 b	0.60 b	10.65 b	277.80 a	0.62 b	0.83 b	0.90 b	1.09 a
180	21.08 a	0.65 a	11.53 a	266.28 c	0.65 a	0.85 a	0.94 a	0.99 b
270	20.45 b	0.62 b	11.42 a	273.58 b	0.62 b	0.83 b	0.92 b	1.06 a
Potassium fertilizer type × FA rate interaction								
Control	18.34 d	0.53 e	9.62 d	295.99 a	0.54 d	0.76 e	0.84 e	1.27 a
CRK × FA90	21.67 b	0.62 bc	11.00 b	274.35 cd	0.63 bc	0.85 b	0.93 b	1.06 bc
CRK × FA180	22.78 a	0.69 a	12.04 a	257.98 e	0.68 a	0.88 a	0.97 a	0.94 d
CRK × FA270	22.15 ab	0.65 b	11.85 a	268.71 d	0.64 b	0.84 bc	0.93 b	1.02 c
KS × FA90	18.69 cd	0.57 d	10.30 c	281.24 b	0.60 c	0.80 d	0.87 d	1.13 b
KS × FA180	19.37 c	0.60 cd	11.01 b	274.58 cd	0.61 bc	0.82 cd	0.92 bc	1.05 bc
KS × FA270	18.77 cd	0.58 d	10.98 b	278.45 bc	0.60 c	0.80 d	0.90 cd	1.08 bc
Source of variance								
Potassium fertilizer type	<0.0001	<0.0001	<0.0001	<0.0001	0.0003	0.0002	0.0002	0.0026
FA rate	0.0116	0.0038	<0.0001	0.0002	0.0099	0.0378	0.015	0.0093
Potassium fertilizer type × FA rate	0.5924	0.2062	0.3497	0.0358	0.1341	0.3972	0.2504	0.4533

Means followed by different lowercase letters in the same column were significantly different based on analyses by ANOVA followed by Duncan’s test (*p* < 0.05). Control, no potassium fertilizer; CRK, polymer-coated potassium chloride; KS, sulfate potassium; *P*_n_, photosynthetic parameters including the net photosynthetic rate; *G*_s_, stomatal conductance; *C*_i_, intercellular carbon dioxide concentration; *T*_r_, transpiration rate; ΦPSII, effective quantum yield of PSII photochemistry; *F*_v_/*F*_m_, primary light energy conversion efficiency; *q*_P_, photochemical quenching coefficient; and *q*_N_, non-photochemical quenching coefficient.

### Content of Soil Available Potassium

**Figure 5:**
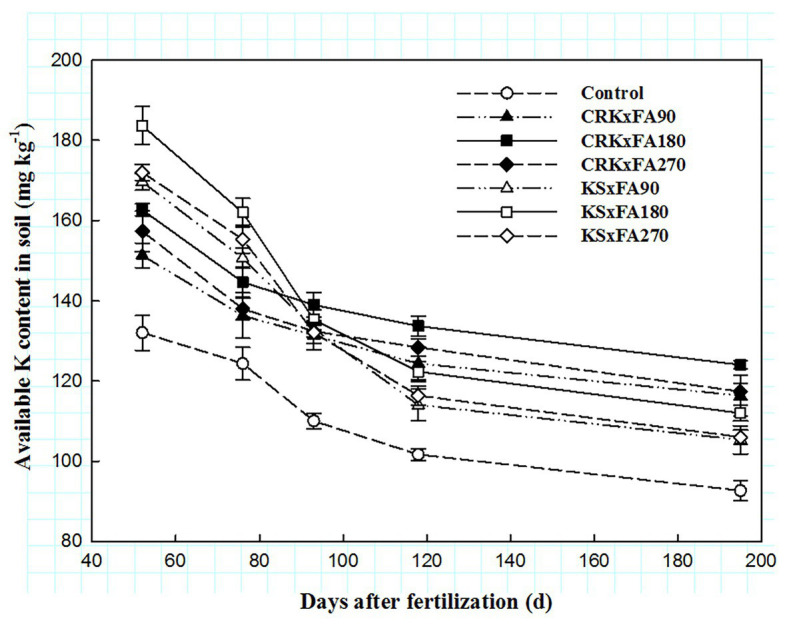
Changes in the soil available potassium content.

In general, the content of soil available potassium in every treatment decreased gradually during the growth period, and the control treatment had the lowest potassium content (Figure 5). Whether combined with CRK or KS, the soil available potassium content of FA180 was higher than those of FA90 and FA270. In addition, the soil available potassium contents of the KS treatments were higher than those of the CRK treatments at the budding and early flowering stages and then showed a downward trend. The soil available potassium contents of the CRK treatments were significantly higher than those of the KS treatments from the early flowering stage to the harvest stage.

### Potassium Uptake and Potassium Use Efficiency

The application of potassium fertilizer and FA significantly increased the potassium uptake of cotton (Table 2). The potassium uptake, KAE, and KRE of the CRK treatments were markedly higher than those of the KS treatments. In addition, the FA180 treatments improved the KAE and the KRE compared with the FA90 and FA270 treatments, regardless of whether they were combined with CRK or KS. There was no significant potassium fertilizer type × FA rate interactive effect on the potassium uptake, KAE, or KRE. In general, the CRK × FA180 treatment resulted in the highest potassium uptake and potassium use efficiency.

**Table 2:** Potassium uptake, potassium agronomy efficiency (KAE), and potassium recovery efficiency (KRE) of cotton plants after harvesting in 2019.

Treatment	Potassium uptake	KAE	KRE
	(kg ha^−1^)	(kg kg^−1^)	(%)
Potassium fertilizer type			
CRK	247.0 a	13.0 a	34.3 a
KS	227.8 b	11.1 b	31.9 b
FA rate (kg ha^−1^)			
90	230.7 b	11.4 b	32.4 b
180	248.2 a	12.6 a	34.0 a
270	233.3 b	11.8 b	32.8 b
Potassium fertilizer type × FA rate interaction			
Control	181.6 d	–	–
CRK × FA90	238.7 b	12.5 b	33.5 b
CRK × FA180	260.0 a	13.6 a	35.3 a
CRK × FA270	242.6 b	12.8 b	34.0 b
KS × FA90	222.2 c	10.4 d	31.3 d
KS × FA180	236.1 b	11.6 c	32.6 c
KS × FA270	224.5 c	10.8 d	31.6 d
Source of variance			
Potassium fertilizer type	<0.0001	<0.0001	<0.0001
FA rate	<0.0001	0.0006	0.0002
Potassium fertilizer type × FA rate	0.1062	0.9636	0.5871

Means followed by different lowercase letters in the same column were significantly different based on analyses by ANOVA followed by Duncan’s test (*p* < 0.05). Control, no potassium fertilizer; CRK, polymer-coated potassium chloride; and KS, sulfate potassium.

### Cotton Yields, Fiber Quality, and Net Profits

The potassium fertilizer type, FA rate and their interaction markedly affected the boll number, boll weight in 2018 and 2019, and the seed cotton yield and lint yield in 2019 (Table 3), and the Control treatments had the lowest values. The yield and yield components of the FA180 treatments were markedly higher than those of the FA90 and FA270 treatments under any potassium fertilizer type. There was no significant difference in lint yield between the FA90 and FA270 treatments. Generally, the CRK treatments improved the boll weight and boll number compared with the KS treatments. Similarly, the seed cotton yield of the CRK treatments also increased. The lint yield changed with the seed cotton yield, and the yield increase effect was consistent. Specifically, the lint yield of the CRK treatments increased by 6.78–9.78% compared with those in the KS treatments. The boll weight and boll number in the CRK × FA180 treatment were the highest of all the treatments. In addition, there was no significant effect on the lint percentage in these treatments.

**Table 3:** Cotton yields and yield components under different treatments during the 2018 and 2019 growing seasons.

Treatment	2018					2019				
	Bolls no.	Boll weight	Seed cotton yield	Lint percentage	Lint yield	Bolls no.	Boll weight	Seed cotton yield	Lint percentage	Lint yield
	(m^2^)	(g)	(kg ha^−1^)	(%)	(kg ha^−1^)	(m^2^)	(g)	(kg ha^−1^)	(%)	(kg ha^−1^)
Potassium fertilizer type										
CRK	81.26 a	6.18 a	5019.10 a	45.66 a	2291.75 a	80.36 a	6.04 a	4940.04 a	45.55 a	2210.17 a
KS	78.71 b	5.97 b	4699.05 b	45.68 a	2146.30 b	77.73 b	5.68 b	4431.00 b	45.58 a	2013.39 b
FA rate (kg ha^−1^)										
90	78.98 b	6.01 b	4744.58 b	45.59 a	2163.07 b	77.68 c	5.76 b	4475.20 c	45.54 a	2037.78 c
180	81.48 a	6.19 a	5041.42 a	45.67 a	2302.52 a	80.65 a	5.98 a	4824.09 a	45.63 a	2200.93 a
270	79.48 b	6.03 b	4791.23 b	45.74 a	2191.48 b	78.80 b	5.84 b	4605.83 b	45.52 a	2096.63 b
Potassium fertilizer type × FA rate interaction										
Control	71.52 e	5.54 f	3963.47 d	45.59 a	1807.12 d	69.22 e	5.21 d	3609.66 e	45.23 a	1632.83 e
CRK × FA90	80.63 b	6.06 cd	4884.05 b	45.57 a	2225.78 b	78.33 cd	5.88 b	4607.12 c	45.53 a	2097.92 c
CRK × FA180	82.96 a	6.26 a	5191.38 a	45.75 a	2375.10 a	82.35 a	6.17 a	5080.23 a	45.59 a	2316.15 a
CRK × FA270	80.27 bc	6.21 ab	4981.89 b	45.65 a	2274.36 b	80.42 b	6.05 a	4869.51 b	45.51 a	2216.43 b
KS × FA90	77.39 d	5.95 de	4605.12 c	45.60 a	2100.36 c	77.03 d	5.63 c	4343.28 d	45.53 a	1977.64 d
KS × FA180	80.25 bc	6.11 bc	4891.46 b	45.59 a	2229.94 b	78.96 c	5.78 b	4567.96 c	45.66 a	2085.70 c
KS × FA270	78.71 c	5.84 e	4600.56 c	45.83 a	2108.59 c	77.24 d	5.62 c	4342.15 d	45.54 a	1976.83 d
Source of variance										
Potassium fertilizer type	<0.0001	<0.0001	<0.0001	0.9059	<0.0001	<0.0001	<0.0001	<0.0001	0.8307	<0.0001
FA rate	<0.0001	0.0017	<0.0001	0.7298	<0.0001	0.0009	0.0001	<0.0001	0.7793	<0.0001
Potassium fertilizer type × FA rate	0.0304	0.0111	0.2528	0.6701	0.4862	0.04679	0.0389	0.0163	0.9714	0.0085

Means followed by different lowercase letters in the same column were significantly different based on analyses by ANOVA followed by Duncan’s test (*p* < 0.05). Control, no potassium fertilizer; CRK, polymer-coated potassium chloride; and KS, sulfate potassium.

The potassium fertilizer types and FA rates improved fiber quality compared with that in the Control treatment (Table 4). However, there was no significant potassium fertilizer type × FA rate interactive effect on the fiber quality in either year. In particular, the FA180 treatments improved the fiber length, uniformity, and strength compared with those in the FA90 and FA270 treatments, but there was no significant effect on the micronaire or fiber elongation. Similarly, the CRK treatments markedly increased the fiber length, uniformity, and strength compared with those of the KS treatments, but the micronaire and fiber elongation were similar. The CRK × FA180 treatment achieved the best performance in terms of fiber quality. There was no significant potassium fertilizer type × FA rate interactive effect on the fiber quality.

**Table 4:** Cotton fiber qualities under different treatments after 2 years of fertilization (in 2019).

Treatment	Fiber length	Fiber uniformity	Micronaire	Fiber elongation	Fiber strength
	(mm)	(%)		(%)	(cN tex^−1^)
Potassium fertilizer type					
CRK	28.3 a	85.2 a	4.8 a	7.1 a	28.2 a
KS	28.0 b	84.3 b	4.8 a	7.1 a	27.6 b
FA rate (kg ha^−1^)					
90	28.1 b	84.5 b	4.8 a	7.1 a	27.6 b
180	28.3 a	85.3 a	4.8 a	7.1 a	28.3 a
270	28.1 b	84.5 b	4.7 a	7.1 a	27.9 b
Potassium fertilizer type × FA rate interaction					
Control	27.2 c	83.7 d	4.7 a	7.1 a	26.2 e
CRK × FA90	28.2 b	84.9 b	4.8 a	7.1 a	27.8 bc
CRK × FA180	28.5 a	85.9 a	4.8 a	7.1 a	28.7 a
CRK × FA270	28.2 b	84.8 bc	4.7 a	7.1 a	28.1 b
KS × FA90	28.0 b	84.0 d	4.8 a	7.1 a	27.4 d
KS × FA180	28.1 b	84.7 bc	4.8 a	7.1 a	27.8 bc
KS × FA270	28.0 b	84.2 d	4.7 a	7.1 a	27.6 cd
Source of variance					
Potassium fertilizer type	0.0004	0.0007	0.7153	0.9034	0.0007
FA rate	0.0222	0.0049	0.4096	0.8956	0.0042
Potassium fertilizer type × FA rate	0.1367	0.3845	0.859	0.9994	0.3426

Means followed by different lowercase letters in the same column were significantly different based on analyses by ANOVA followed by Duncan’s test (*p* < 0.05). Control, no potassium fertilizer; CRK, polymer-coated potassium chloride; and KS, sulfate potassium.

The mean annual revenue, costs, and net profits from the different treatments in 2018 and 2019 were calculated, and the control had, by far, the lowest value (Table 5). The net profit of the FA180 treatment was higher than those of the FA90 and FA270 treatments. Compared with the KS treatments, the CRK treatments markedly improved the net profit. The net profit from the CRK × FA180 treatment was the highest in both years and was 81 and 156% higher in 2018 and 2019, respectively, compared with that of the control treatment. Overall, the CRK × FA180 treatment was the best in terms of the cotton yield, fiber quality, and economic benefit.

**Table 5:** Mean annual revenues, costs, and net profits from potassium treatments (2018 and 2019).

Treatment	Total revenue		Fertilizer costs	Labor cost	Other costs	Net profit		Change vs. Control (%)	
	2018	2019	($ ha^−1^ year^−1^)			2018	2019	2018	2019
Control	5,340	4,863	190	3,000	721	1,429	953	–	–
CRK × FA90	6,580	6,207	368	3,300	721	2,191	1818	53	91
CRK × FA180	6,994	6,844	387	3,300	721	2,586	2,436	81	156
CRK × FA270	6,712	6,560	407	3,300	721	2,284	2,133	60	124
KS × FA90	6,204	5,851	403	3,300	721	1780	1,427	25	50
KS × FA180	6,590	6,154	423	3,300	721	2,147	1711	50	80
KS × FA270	6,198	5,850	442	3,300	721	1736	1,387	21	46

Mean prices of fertilizers and other non-labor expenses in China (United States dollars per metric ton): polymer-coated potassium chloride $485.6, potassium sulfate $540.1, calcium superphosphate $149.3, urea $238.8, and cotton $1347.2. Other costs included machinery, plastic film, irrigation, pesticides, insecticides, seeds, and other materials and services. Mean cost of labor in China: $12.80 for one employee/day/ha. Control, no potassium fertilizer; CRK, polymer-coated potassium chloride; and KS, sulfate potassium.

## Discussion

### Leaf Photosynthesis in Cotton

Premature senescence caused by potassium deficiency has become one of the main factors limiting high and stable cotton yields in China (Hu et al., 2016). The application of potassium fertilizer promotes the absorption of nitrogen and phosphorus, enhances the physiological activity of leaves, prolongs the functional period of the plant, and prevents premature aging to maintain a higher photosynthetic rate and provide sufficient carbohydrates for subsequent cotton growth and development (Tsialtas et al., 2016). As a water-soluble component of HA, FA has a low molecular weight; therefore, it more easily promotes the absorption of potassium by plants. At the same time, the carboxyl group and phenolic hydroxyl group of FA react with the amide group of potassium to form a complex (Farid et al., 2018). In this study, the values of SPAD, chlorophyll fluorescence and photosynthetic parameters in the FA180 treatment were higher than those in the FA90 and FA270 treatments. It should be noted that the SPAD-502 device does not give the chlorophyll concentration in the leaf but only unitless estimates that we used to compare responses to treatments and environmental conditions (Escobar-Gutiérrez and Combe, 2012). Readers should bear in mind that the accuracy of SPAD values decreases for readings over 50, due to the asymptotic relationship between leaf transmittance and chlorophyll content (Escobar-Gutiérrez and Combe, 2012). Our results must be regarded under this caveat. In general, based on its higher photosynthetic parameters, the CRK × FA180 treatment delayed leaf senescence. The net photosynthetic rate (*P*_n_) and stomatal conductance (*G*_s_) rate increased, and the intercellular carbon dioxide concentration (*C*_i_) rate decreased due to the improvement of plant photosynthesis, which required more carbon dioxide (Yang et al., 2017). The physiological activity of the leaves in the KS treatments was decreased, with the lower leaves prone to losing green and the leaf margins appearing mostly yellow and withered. The leaves showed premature senescence, which reduced the green photosynthetic area, affected the growth of the plants, and ultimately caused the yield and quality of the cotton to decline. This result is similar to the findings of Kong et al. (2016).

### Soil Available Potassium

The level of soil available potassium is closely related to the cotton yield and premature aging. Cotton has a straight root system. The number of roots is relatively low, the absorption capacity of potassium from soil is low, and cotton is more sensitive to potassium deficiency compared to other field crops (Raper, 2018). Meanwhile, FA can reduce the absorption and fixation of potassium in soil and improve the utilization rate of available potassium (Tan, 1978). In this study, compared with the FA90 and FA270 treatments, the FA180 treatments increased the soil available potassium content, indicating that only an appropriate FA rate had a positive impact on the soil potassium. The appropriate FA application promoted the release of insoluble potassium, and increasing the quantity of available potassium can alleviate the adverse effects of potassium fertilizer on soil and crops and improve the crop quality (Xu et al., 2012). In addition, due to the continuous release of potassium from the CRK, the content of soil available potassium increased significantly from the full boll-setting stage to the harvest, which was similar to the results of Tian et al. (2017). The application of CRK had better effects than did applying KS, mainly because CRK changed the physical properties of the conventional potash fertilizer, thus delaying the dissolution rate of available potassium in the soil and reducing leaching and loss (Yang et al., 2017). The CRK × FA180 treatment increased the available potassium in the soil during the cotton flowering and boll-formation stages, when the demand for potassium is highest.

### Potassium Uptake and Potassium Use Efficiency

Many parameters can be used to describe fertilizer use efficiencies, and the key to improving the potassium use efficiency lies in the potassium uptake. In the present research, the parameters KAE and KRE were used. Regardless of how much FA was applied, the potassium uptake, KAE and KRE of the CRK treatments were markedly higher than those of the KS treatments, which may have been due to the high potassium uptake. Similar results were also found by Chen et al. (2020). In addition, the FA application rate significantly affected the potassium uptake, KAE and KRE. The values of KAE and KRE in the FA180 treatments were higher than those in the FA90 and FA270 treatments. Thus, the application of FA can improve the potassium use efficiency and promote potassium absorption by cotton (Staunton and Roubaud, 1997).

### Yield and Fiber Quality of Cotton

The effects of potassium on cotton yields come from many aspects that are directly reflected in some characteristics and yield components, including the density, boll number, boll weight, and lint percentage (Read et al., 2006). FA accelerates plant metabolism, strengthens photosynthesis, and increases sugar and dry matter accumulation to improve the crop resistance to freezing, disease, and other stresses and to improve the crop yield (Ohno et al., 2009; Li et al., 2018). In this study, the FA180 treatments significantly increased the boll number and boll weight, resulting in higher seed and lint cotton yields than those under the FA90 and FA270 treatments, but there were no significant differences in the lint percentage. In addition, the release characteristics of CRK provided sufficient potassium for the whole growth period of cotton and improved the seed cotton yield, which was higher than that in the KS treatments. Similar results were reported by Yang et al. (2016). There was a positive interaction between the potassium fertilizer type and FA rate in terms of the cotton boll number and boll weight in 2018 and 2019, and the seed and lint cotton yields were also markedly affected by this interactive effect in 2019. In addition, the net profit of CRK × FA180 markedly increased compared with those of the other fertilization combinations due to its high-yields.

Many studies have indicated that potassium application improves the cotton fiber quality (Tariq et al., 2018; Zhao et al., 2019). The application of FA improves resistance and immunity in plants, inhibits the growth and reproduction of harmful organisms in plants, maintains the dominant advantage of beneficial bacteria in plants, and enhances the absorption and transformation of nutrients to improve the crop quality (Rauthan and Schnitzer, 1981). In this study, compared with the low- and high-FA treatments, the moderate-FA treatments increased the fiber length, uniformity, and strength, but there was no significant difference in the fiber elongation or micronaire value. Compared with the KS treatments, the CRK treatments increased the fiber length, uniformity, and strength, which may have been due to the continuous sufficient supply of potassium in the critical growth period. Furthermore, the effects of the potassium fertilizer type and FA rate on cotton fiber elongation and micronaire were not significant, and the effects of genetic regulation on fiber elongation might be greater than the effects of fertilization (Li et al., 2005).

## Conclusion

The potassium fertilizer type, FA rate and their interactions had significant effects on cotton leaf photosynthesis, yield, and potassium use efficiency. The soil available potassium content was improved by CRK due to the continuous release of potassium nutrients, and FA180 also supported potassium uptake. The CRK × FA180 treatment increased the cotton potassium uptake, KAE, and KRE. In addition, the net profit in the CRK × FA180 treatment was also increased by 13.22–48.96% in 2018 and 14.21–75.63% in 2019 compared with those under the other potassium treatments. Thus, CRK in combination with 180 kg ha^−1^, FA is suggested for cotton fertilization.

## Data Availability Statement

The original contributions presented in the study are included in the article/Supplementary Material, further inquiries can be directed to the corresponding author.

## Author Contributions

JG and XY conceived and designed the experiments and wrote the manuscript. JC, QL, and XH analyzed the data. SL and HL were involved in the related discussion. YL helped to improve the quality of the manuscript. All authors contributed to the article and approved the submitted version.

### Conflict of Interest

JC was employed by the Kingenta Ecological Engineering Group Co., Ltd.The remaining authors declare that the research was conducted in the absence of any commercial or financial relationships that could be construed as potential conflict of interest.
